# Sharp Sticks

**Published:** 1865-10

**Authors:** A. Lawrence


### "SHARP STICKS."

BY A. LAWRENCE.

In the last number of the "Register" (p. 330) appears what was evidently intended as an annihilating criticism upon what I have heretofore said on steam pressure in vulcanizers, and the steam guage. My professional brother has the undoubted right to express his opinion upon the subjects just named, or any other; but I submit, that when he attempts to review the writings of another, he ought, out of regard to truth and the rights of others, never to resort to the expedient of jumping at such conclusions as a fair reading would fail to justify.

Nor, do I think my castigator exhibits the most refined taste in making quotations from others and attributing them to me, as he has done, for the purpose of making me appear ridiculous and himself very "sarkastikal," as the saying is.

He commences by giving me what I never claimed, *i. e.* credit for "a valuable suggestion regarding the strength that vulcanizers should possess."

This, I consider, as similar to the sliming a fat buck receives from the Boa Constrictor, preparatory to the interesting operation of being swallowed whole.

Oh! conscience, what a savory morsel I should be in the insatiate maw of my dear friend, should I ever reach that delectable haven of rest.

Now, as I intend to be very brief in this matter, I will only allude to the *sharp hits.* In relating an accident he unfortunately had with his "old fashioned vulcanizer," we are triumphantly assured that it "would not have been averted a jot by the use of an $18,00 steam guage" Further along he gives the astounding information that neither the thermometer nor the guage alone are "at all safe" and that "the guage must be watched just as closely as the thermometer." We do not differ in opinion so far. I gave my preference to the guage, because I thought *it* less liable to accidents, and therefore safer and more convenient than the thermometer, but I never expressed the idea that it gave any additional strength to the vulcanizer, or that any less watching would be required, and none but a very "superficial man" could so render anything I have written upon the subject, unless aided by *some spirit* not considered within the pale of professional comity.

The next turn of the screw accounts for my "refreshing security," which I flatter myself arose from feeling that with the guage there were no mercury tubes and bulbs to be broken, &c., but I am undeceived by learning that it is familiarity in "a certain instance," though left in the dark as to what instance, with "a small steam engine." I am truly thankful for the information given but must deny publishing any article in which the quoted words appear. They belong to Dr. A. T. Johnson, (Register for May & June—p. 194) and I am no more responsible for their appearance, than my tormentor is for being made a "little lower than the angels." I deny ever having manufactured an article with the title his other quotations gives me credit for, and which he says is vulgarly called amalgam, and such license forces me to the conviction that vulgarity is not confined to that article. For the information which I think due the great advocate of safety-valves, &c., I now say, that I have some slight acquaintance with "a small steam engine" which I use in the manufacture of "Lawrence's Amalgam" the best in the market—only $3,00 per oz. and for sale at the Dental Depots.

I beg also to suggest, (respectfully of course,) that the writer of the article in question is not very well posted in steam guages, inasmuch as he seems not to be aware of the fact, that the one I have taken the liberty to mention, has no "piston," as he states. The piston guage went out of general use years ago, *because* it was "a complicated machine," and unlike those manufactured at this time by the American Steam Guage Co.

Of the writer of the article, to which I take the foregoing exceptions, I unfortunately know nothing, but presume him to be really a capital fellow; and I beg to assure him, if he will only tolerate the same liberty of opinion in others that he claims for himself, there will be no further occasion for the use of *sharp sticks.*

